# Genetic aberrations of *NLRC5* are associated with downregulated MHC‐I antigen presentation and impaired T‐cell immunity in follicular lymphoma

**DOI:** 10.1002/jha2.116

**Published:** 2020-10-14

**Authors:** Jay Gunawardana, Justina N. Lee, Karolina Bednarska, Valentine Murigneux, Lilia Merida de Long, Muhammed B. Sabdia, Simone Birch, Joshua W. D. Tobin, Maher K. Gandhi

**Affiliations:** ^1^ Mater Research University of Queensland, Translational Research Institute Brisbane Queensland Australia; ^2^ Diamantina Institute University of Queensland Brisbane Queensland Australia; ^3^ QFAB Bioinformatics Institute for Molecular Bioscience University of Queensland Brisbane Queensland Australia; ^4^ Princess Alexandra Hospital Brisbane Queensland Australia

**Keywords:** antigen presentation, hematological malignancies, immunotherapy, non‐Hodgkin lymphoma

## Abstract

Follicular lymphoma (FL) is the most common indolent non‐Hodgkin lymphoma. Twenty to twenty‐five percent of FL patients have progression of disease within 24 months. These patients may benefit from immunotherapy if intact antigen presentation is present. Molecular mechanisms impairing major histocompatibility complex class‐I (MHC‐I) in FL remain undefined. Here, by sequencing of 172 FL tumours, we found the MHC‐I transactivator NLRC5 was the most frequent gene abnormality in the MHC‐I pathway. Pyrosequencing showed that epigenetic silencing of the *NLRC5* promoter occurred in 30% of cases and was mutually exclusive to copy number loss (CNL) in *NLRC5* (∼6% of cases). Hypermethylation and CNLs (“*NLRC5* aberrant”) had reduced *NLRC5* gene expression compared to wild‐type (WT) cases. By NanoString, there was reduced gene expression of the MHC‐I pathway in aberrant tissues, including immunoproteasome components (*PSMB8* and *PSMB9)*, peptide transporters of antigen processing (*TAP1*), and MHC‐I (*HLA‐A*), compared to WT. By immunofluorescent microscopy, fewer NLRC5 protein‐expressing malignant B‐cells were observed in NLRC5 aberrant tissue sections compared to NLRC5 WT (*P *< .01). Consistent with a pivotal role in the activation of CD8^+^ T‐cells, both *CD8* and *CD137* strongly correlated with NLRC5 expression (both *r *> 0.7; *P *< .0001). Further studies are required to determine whether patients with aberrant NLRC5 have a diminished response to immunotherapy.

## 1 INTRODUCTION

Follicular lymphoma (FL) is the most common indolent non‐Hodgkin lymphoma (NHL), accounting for 25‐30% of all NHL cases [1, 2, 3]. It is characterised by a proliferation of neoplastic germinal center B‐cells with a follicular pattern [4, 5]. Although FL is responsive to initial therapy, it is considered incurable. Improvements in the treatment of FL disease (notably, the combination of anti‐CD20 antibodies to chemotherapy or “immunochemotherapy”) have increased median survival to over 15 years [6]. However, outcomes are highly heterogeneous, with approximately 20%‐25% having progression of disease within 24 months (POD24). These patients have mortality rates of up to 50% with conventional therapies in the 5 years after the POD defining event [7]. In others, FL will transform to an aggressive subtype (t‐FL), a therapeutically challenging scenario that is poorly responsive to high‐dose chemotherapy and is associated with genetically mediated acquired immune escape [8]. In the remainder of patients, multiple relapses will occur over a patient's lifetime, with the patient having remissions of progressively shorter duration to each course of immunochemotherapy. In all these patient groupings (POD24, t‐FL, and the multiple relapsed), new approaches are required, with new agents currently in clinical trials. Options include agents that engage the host antitumoural immune response, such as lenalidomide and checkpoint inhibitors (CPIs) [9].

Tumours use multiple immune evasion strategies to establish an immunosuppressive microenvironment. A number of such strategies have already been described in FL that spotlight the importance of the interplay between the malignant genetic landscape and host immunity in FL [10]. Mutations (eg, in *TNFSRF14* and *CTSS*) have been described that promote lymphomagenesis through various cell‐extrinsic mechanisms, including recruitment of CD4^+^ T‐follicular helper cells, to foster a supportive microenvironment [11, 12, 13]. Importantly, mutations have also been found to impair antigenicity: that is, the ability of the FL B‐cell to present antigen to a T‐cell in the context of a peptide‐major histocompatibility complex (MHC) complex. Tumours, which lose MHC expression or acquire upstream defects in antigen presentation, will be relatively resistant to immune‐mediated elimination by tumour‐specific T‐cells, resulting in impaired activation of CD4^+^ (MHC‐II recognition) and CD8^+^ T‐cells (MHC‐I recognition). It is likely that such tumours will be less prone to respond to CPIs. New genetic biomarkers that can identify patients who have reduced antigenicity and hence would be less likely to benefit from CPI remains an unmet clinical priority as these patients might be better considered for alternate treatment options.

In FL, lack of antigenicity is best typified by cyclic‐AMP response element binding protein (CREBBP) [14]. Mutations in *CREBBP* confer inferior progression‐free survival (PFS), suggesting that these mutated clones assist FL to survive first‐line immunochemotherapy. It achieves this via downstream effects on decreased antigen presentation. Specifically, its mutations are characterized by reduced transcript and protein abundance of MHC‐II on FL B‐cells. This is in keeping with the known role of *CREBBP* in promoting MHC‐II transactivator (CIITA)‐dependent transcriptional activation, which serves as a mechanism to evade host CD4^+^ T‐cell immunity. Importantly, *CREBBP* mutant B‐cells stimulate less T‐cell proliferation compared to wild‐type (WT) B‐cells from the same tumour. Downstream of *CREBBP*, loss‐of‐function mutations in *CIITA* resulting in diminished MHC‐II surface expression have also been reported in aggressive malignant lymphoma [15], suggesting the MHC‐II/CD4^+^ T‐cell axis disruption is an important mechanism to induce loss of antigenicity.

The T‐cell receptor (TCR) of cytotoxic CD8^+^ T‐cells has the potential to bind to neoantigens presented within the context of MHC‐I molecules present on FL B‐cells. Downregulation of MHC‐I molecules has been found in approximately 20‐60% of common solid malignancies [16]. Moreover, changes in MHC expression correlate with the clinical course in several malignancies [16, 17]. In aggressive NHLs, mutations in *β2M* [18] and *EZH2* have been identified as mechanisms by which MHC‐I is aberrantly expressed [19]. However, no comprehensive analysis of the MHC‐I pathway in indolent NHL has been undertaken to date.

NLRC5 (also known as CITA) is a key transcription coactivator of genes involved in the MHC‐I pathway, including *HLA‐A, HLA‐B, HLA‐C*, *β2M*,*PSMB8, PSMB9, TAP1*, and *TAP2* [20, 21]. Loss of NLRC5 function leads to downregulation of MHC‐I‐related genes. This in turn impairs subsequent tumour antigen presentation for cytotoxic T‐cell‐mediated killing, a deficiency exploited by cancer cells to thrive in an immunocompromised milieu. Recently, genetic aberrations in NLRC5 (including promoter methylation, copy number loss [CNL], and somatic mutations) were shown to downregulate genes in the MHC‐I antigen‐presentation pathway in 16 solid cancers [22]. The frequency of the genetic aberration type varied considerably by tumour type; however, irrespective of the genetic mechanism, NLRC5 gene expression was reduced and low expression was consistently significantly associated with inferior patient survival. To date, NLRC5 aberrations have yet not been reported in any indolent lymphoid malignancy.

In this study, we demonstrate that NLRC5 genetic and epigenetic aberrations lead to reduced NLRC5 RNA and protein expression, downregulation of MHC‐I antigen presentation genes, and functional impairment of cytotoxic T‐cell and T‐cell activation markers in FL patient tissue. Our data indicate that NLRC5 is a novel MHC‐I‐related immune evasion mechanism utilized by FL tumours to impair antigenicity.

## 2 MATERIALS AND METHODS

### 2.1 Patient samples and nucleic acid extraction

The study included 172 FL pretherapy formalin‐fixed paraffin embedded (FFPE) tissue biopsies obtained from the lymphoma repository at Princess Alexandra Hospital (Brisbane, Australia). Median follow‐up was 5.1 years. Patient characteristics are provided in Table 1. Only *de‐novo* cases were included. Excluded were biopsies of patients with relapsed or transformed disease. Peripheral blood was available from four patients. Nucleic acids were extracted using the AllPrep DNA/RNA FFPE Kit (Qiagen). Control lymph node samples were sourced from 16 surgically excised lymph nodes without histological evidence of malignancy and prepared similarly.

**TABLE 1:** Patient characteristics

Variable		N	%
Study cohort		172	100
Median age (range)			61.3 (23‐86) years
NLRC5	WT	116/172^a^	67.4
	SNV	11/172	6.4
	CNL	11/172	6.4
	Methylated	39/132	30
Sex	Male	85/172	49.4
Ann Arbor stage	I‐II	37/172	21.5
	III‐IV	135/172	78.5
ECOG	>1	13/172	7.6
FLIPI	1‐2	96/172	55.8
	3	75/172	43.6
Treatment	No treatment	20/172	11.6
	R‐CHOP	79/172	45.9
	R‐CVP	37/172	21.5
	B‐R	13/172	7.6
	Other^b^	6/172	3.5
	Radiation alone	17/172	9.8
POD24		40/172^c^	23.2
Extranodal site involvement		62/172^d^	36.0
Bone marrow involvement		76/172^d^	44.2
Presence of B symptoms		35/172^d^	20.3
Median follow‐up		5.1 years	

Abbreviations: B, bendamustine; CHOP, cyclophosphamide, doxorubicin hydrochloride, vincristine and prednisolone; CNL, copy number loss; CVP, cyclophosphamide, vincristine sulfate, and prednisone; ECOG, Eastern European Oncology Group; FLIPI, Follicular Lymphoma International Prognostic Index; POD24, progression of disease within 24 months.; R, rituximab; SNV, single nucleotide variant; WT, wild type.

^a^ Methylation status not known in 40 cases.

^b^ CHOP + Etoposide, R‐Chlorambucil, R‐Monotherapy.

^c^ Missing data for 10 cases.

^d^ Missing data for one case.

### 2.2 Targeted sequencing

MHC‐I antigen presentation pathway gene sequencing was performed using the SureSelect XT™ (Agilent Technologies) hybrid capture‐based target enrichment methodology as part of a previously published FL study [23]. Coverage was > 400x. Reads were mapped to the hg19 human genome with Novoalign v3.02.08 (Novocraft), duplicates reads were removed using Picard v1.131, indel realignment and base quality score recalibration were applied using GATK v3.4. The mutations (single nucleotide variants [SNVs] and small indels) were called by two softwares: MuTect2 v3.6 and Lofreq v2.1.2 and subsequently filtered variants were identified by both programs. SnpEff v4.1 was used to annotate and predict the effect of the variants using Ensembl annotation (GRCh37, release 75) and only included variants predicted to have a high or moderate impact on the protein. Variants were excluded if they: (a) were present in the Genome Aggregation database (exomes release 2.0.2) at a frequency more than 1%; or (b) were present in any of the 16 control lymph node samples; or (c) were identified with a variant allele frequency less than 5%. Genome copy number alterations were called using ONCOCNV (v6.6), CNVPenalizer (v1.4), and Bedtools (v2.21.0), and reported if in agreement with all three tools.

### 2.3 Quantitative genomic PCR

Biological validation of the bioinformatically predicted 11 cases with *NLRC5* copy number variations was done by quantitative genomic PCR (absolute quantification method) and compared to 11 *NLRC5* copy‐number neutral cases using 12.5 ng of input DNA, SYBR green reagent, and the following forward and reverse intronic primers, respectively:

5′‐TCGTGGGTCACACTTTCATC‐3′ and 3′‐GTGGGTTTTCTGTTGGCATT‐5′.

### 2.4 Pyrosequencing

The *NLRC5* promotor has a CpG island of ∼578 bp starting at position –278. Two hundred to four hundred nanograms of DNA from 132 FL cases were bisulfite converted and PCR amplified to generate biotin labeled double‐stranded amplicons. Pyrosequencing reactions were run on single‐stranded DNA templates using three pyrosequencing primers covering 19 CpG sites on a PyroMark Q24 and the methylation percentage was obtained for each site. An unmethylated DNA control was run with each conversion to test bisulfite conversion integrity. If methylation of a given CpG site was greater than the mean methylation of 21 healthy controls, that site was considered methylated. If more than half of CpG sites interrogated were methylated, the sample was considered methylated.

### 2.5 Digital transcriptomic analysis (NanoString)

Extracted RNA from 29 FL tumours was run on a customised NanoString codeset to quantitate digital multiplexed gene expression [24, 25, 26, 27] in the following comparison groups: (a) *NLRC5* WT (non‐CNL/non‐methylated/non‐SNV cases, n = 7) versus *NLRC5* SNV cases (n = 8); (b) *NLRC5* WT (non‐CNL/non‐methylated/SNV cases, n = 9) versus *NLRC5* aberrated (10 *NLRC5* CNL and 8 *NLRC5* promotor methylated, n = 18). The panel comprised the following genes: immune effectors and activation markers (*CD4, CD8A, CD56, CD58, CD68, CD163*, and *CD137*) and MHC‐I‐related genes (*NLRC5, β2M, HLA‐A, HLA‐B, HLA‐C, HLA‐DRB1, HLA‐DRA, PSMB8, PSMB9, TAP1*, and *TAP2*). Resulting gene counts were normalised to housekeeping genes (*GAPDH, OAZ1, PGAM1*, and *PGK1*) using nSolver 4.0 software.

### 2.6 Multispectral immunofluorescent microscopy

Immunostaining for NLRC5 and CD20 was performed using the Opal^®^ 4 lymphocyte kit (Perkin Elmer) on four NLRC5 WT and three NLRC5 aberrated (two NLRC5 CNL and one NLRC5 promotor methylated) cases using 1 μm tissue sections mounted on superfrost microscope slides. Image acquisition was performed on the Vectra^®^ microscope (Perkin Elmer) acquiring five random regions‐of‐interest for each sample at 20x resolution. Analysis was performed using the Perkin Elmer Inform^®^ 2.1 software. Phenotypes were assigned to segmented cells using supervised computer‐learning algorithms, as published previously [23]. Each phenotype was trained manually by selecting 50 cells representative of the phenotype of interest before the remaining cells were algorithmically assigned a phenotype. Inform software automatically derives cell counts as a percentage of total segmented cells and the mean total fluorescence intensity for each marker. Representative images were captured for visualisation using the FV3000 confocal laser scanning microscope and fv30i software (Olympus).

### 2.7 Statistical analysis

Comparisons between groups were performed using a Pearson correlation or Mann‐Whitney rank sum test, as appropriate. Multiple hypothesis testing was performed using the Holm‐Sidak method (GraphPad Prism 7). Survival analysis was performed with the Kaplan‐Meier method and curves were compared with the log‐rank (Mantel‐Cox) test. Overall survival (OS) was defined as the time from diagnosis to last follow‐up or death from any cause. PFS was defined as the time from diagnosis until progression, relapse, transformation, or death from any cause. Failure‐free survival (FFS) was measured from the start of therapy to the date of disease progression, relapse, or disease‐ or treatment‐related death. Time to second treatment (TT2T) was calculated from time of disease diagnosis to start of second treatment.

## 3 RESULTS

### 3.1 *NLRC5* genetic aberrations are frequent in FL tumours

**FIGURE 1:**
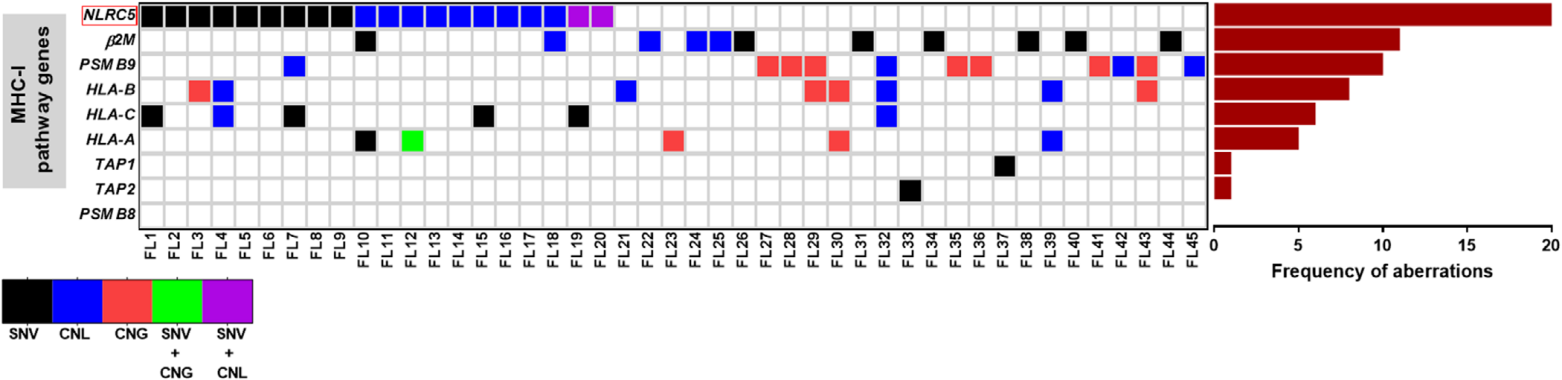
Mutations and copy number alterations in genes in the MHC‐I antigen presentation pathway in 172 diagnostic FL patient tumours. Heatmap (left) and frequency (right) of single nucleotide variants (SNVs), copy number losses (CNLs), and copy number gains (CNGs) for each gene in the pathway found by targeted sequencing. Only aberrant cases are shown.

To characterise MHC‐I antigen presentation deficiencies in FL, we designed a targeted gene sequencing panel covering MHC‐I pathway transcription coactivators, genes coding for immunoproteasome components, peptide transporters of antigen processing, and MHC‐I subunits. DNA from 172 diagnostic FL tumours was tested. Nine genes in the MHC‐I pathway were screened, focussing on copy number alterations (ie, CNLs or gains) and SNVs (Figure 1). *NLRC5* genetic aberrations were the most frequent (in 20 of 172 cases; 11.6%). This was followed by *β2M* (in 11 of 172 cases; 6.4%), *PSMB9* (in 10 of 172 cases; 5.8%), *HLA‐B* (in 8 of 172 cases; 4.7%), *HLA‐C* (in 6 of 172 cases; 3.5%), *HLA‐A* (in 5 of 172 cases; 2.9%), *TAP1* (in 1 of 172 cases; 0.6%), *TAP2* (in 1 of 172 cases; 0.6%), and no aberrations were seen in *PSMB8*.

When only CNLs were interrogated, CNL in *NLRC5* was the most frequently observed CNL and with the exception of 1/11 cases, CNL in *NLRC5* was mutually exclusive of the occurrence of CNL in other components of the MHC‐I pathway. As NLRC5 is known to be an important regulator of this pathway and abnormalities of this gene were the most frequently observed, *NLRC5* was focussed on for subsequent analyses.

### 3.2 SNVs in *NLRC5* are germline variants

After exclusion of variants found in any of the16 healthy controls and variants that result in synonymous amino acid substitutions, we found 12 *NLRC5* coding‐sequence SNVs in our cohort with a single case harbouring more than one variant (11 of 172 cases, 6.4%; Figure S1). SNVs were overwhelmingly missense variants (11 of 12, 91.6%) and clustered predominantly in the leucine‐rich repeat and the atypical caspase activation and recruitment (uCARD) domains of the protein (Figure S2A), similar to observations in solid tumours [22]. However, unlike the mutations reported in solid malignancies, these variants were also detected in matched peripheral blood DNA confirming them as germline variants (ie, not somatic mutations; Figure S2B).

### 3.3 Promotor hypermethylation is the most frequent mechanism of *NLRC5* gene aberration in FL

**FIGURE 2:**
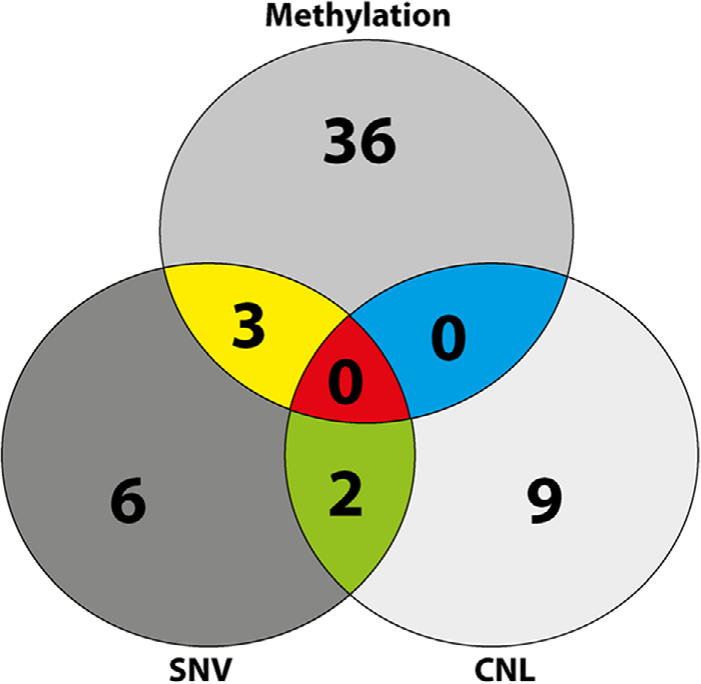
Methylation is the most frequent *NLRC5* genetic abnormality in FL. Venn diagram showing numbers of FL cases with *NLRC5* SNV, CNL, and promotor hypermethylation. All 172 cases underwent targeted sequencing and 132 cases had pyrosequencing done.

Epigenetic changes regulate gene expression that enable malignant cells to escape host immunity [28]. Hypermethylation of the *NLRC5* promotor and consequent gene expression changes have been reported in a spectrum of solid tumours [22]. We sought to test the prevalence of increased methylation at CpG sites in the *NLRC5* promotor by performing pyrosequencing in a subset of our cohort. Promotor hypermethylation was observed in 30% of tumours (39 of 132 cases) indicating that this mechanism is the dominant genetic abnormality in FL compared to CNLs or SNVs (Figure 2).

### 3.4 Aberrant *NLRC5* is associated with reduced *NLRC5* gene expression

**FIGURE 3:**
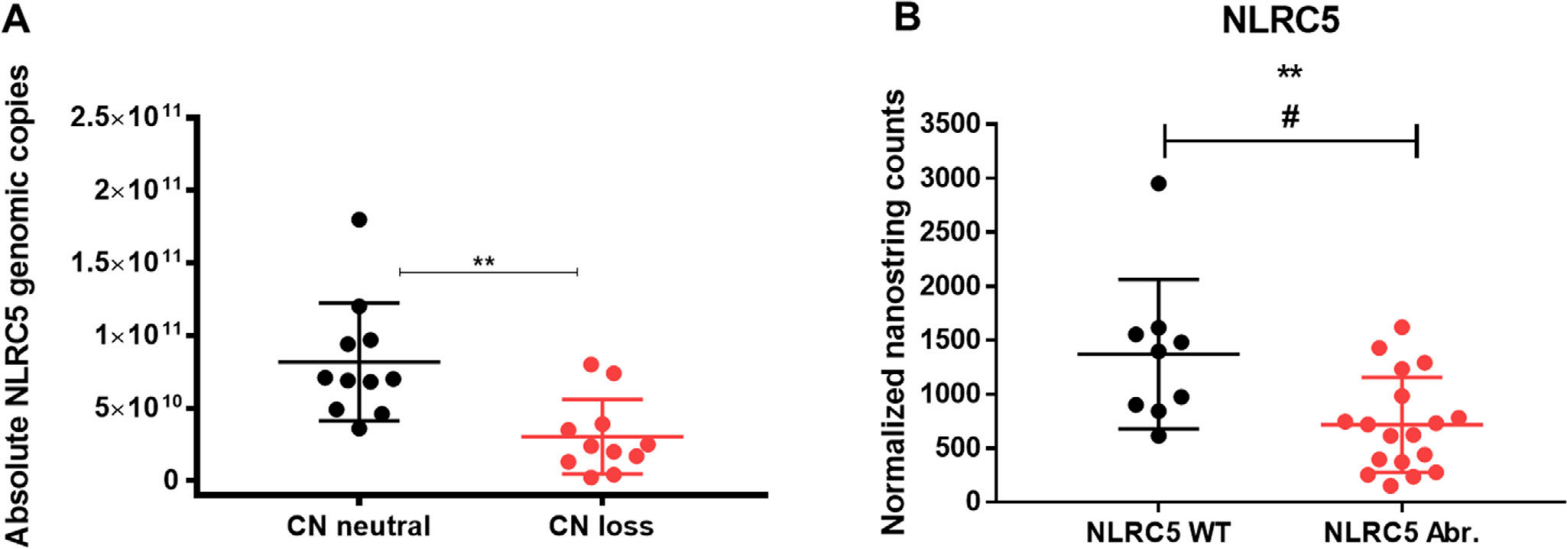
*NLRC5* copy numbers and gene expression in FL tumours stratified by *NLRC5* genetic status. A, Genomic quantitative PCR validation of the 11 cases harbouring *NLRC5* copy number (CN) losses compared to 11 *NLRC5* CN neutral cases. B, As no significant difference in gene expression of CNL versus promotor methylated was observed, cases were combined as “*NLRC5* aberrant.” These showed reduced gene expression compared to wild‐type (WT). Mann‐Whitney test **P < .01; multiple hypothesis tested adjusted ^#^ *P *< .05. Error bars, mean with SD.

All 11 cases (100%) with *NLRC5* copy‐number alterations had a mono‐allelic loss (CNL) of the gene. These findings were validated by quantitative PCR, confirming the observed CNLs in these cases are significant compared to cases that are copy number neutral (*P *< .01; Figure 3A). Digital transcriptomic (NanoString) counts were generated using extracted RNA from 29 FL tumours. As expected, both *NLRC5* germline SNV and WT cases showed similar *NLRC5* gene expression (mean counts 1225 and 1351, respectively, *P *= NS; Figure S2C). However, *NLRC5* gene expression in CNL and hypermethylated cases was significantly reduced in comparison to *NLRC5* WT cases (715 vs 1370, *P *< .01, adjusted *P *< .05; Figure 3B). *NLRC5* genetic (CNL) or epigenetic (methylation) aberrations are collectively referred to as “*NLRC5* aberrations” here onward. Collectively, the lack of association of impaired gene expression with SNV and the reduction in *NLRC5* gene expression with *NLRC5* aberration is consistent with our earlier findings outlined in the Venn diagram (Figure 2). This showed that SNV overlaps with *NLRC5* aberrations, whereas CNL and methylated cases were mutually exclusive of each other.

### 3.5 Aberrated *NLRC5* downregulates MHC‐I antigen presentation pathway genes

**FIGURE 4:**
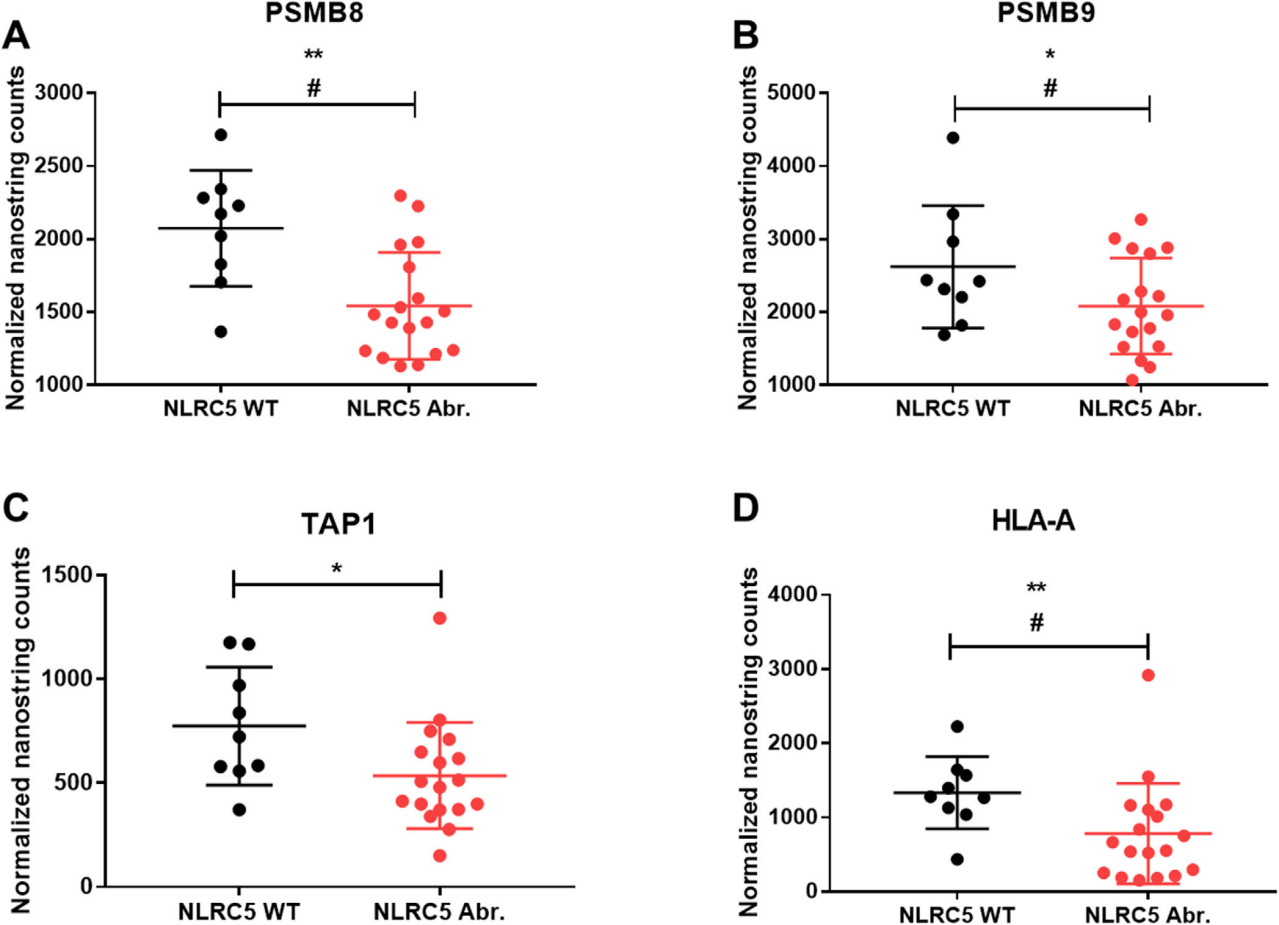
Quantification of MHC‐I pathway genes in FL tumours with *NLRC5* aberrations compared to wild‐type. Expression of MHC‐I pathway genes quantified by NanoString, stratified by *NLRC5* wild‐type (WT) and methylation/copy number loss (Abr.) status. (A) *PSMB8*, (B) *PSMB9*, (C) *TAP1*, and (D) *HLA‐A*. *P < .05; **P < .01; multiple hypothesis tested adjusted ^#^ *P *< .05. Error bars, mean with SD.

Gene expression was quantified to determine if *NLRC5* aberrations affect downstream genes in the MHC‐I antigen presentation pathway. There was reduced expression of MHC‐I antigen presentation pathway genes, including immunoproteasome components (mean *PSMB8*: 1542 vs 2542, *P *< .01, adjusted *P *< .05 and *PSMB9*: 2082 vs 2620, *P *< .05, adjusted *P *< .05; Figure 4A and 4B), peptide transporters of antigen processing (*TAP1*: 536 vs 774, *P *< .05, adjusted *P *= NS; Figure 4C and *TAP2*: 1106 vs 1408, *P *= NS, adjusted *P *= NS), and MHC‐I (*HLA‐A*: 781 vs 1330, *P *< .01, adjusted *P *< .05; Figure 4D, *HLA‐B*: 87 548 vs 101 202, *P *= NS, adjusted *P *= NS; *HLA‐C*: 9419 vs 9470, *P *= NS, adjusted *P *= NS; *β2M*: 69 876 vs 76 032, *P *= NS, adjusted *P *= NS) compared to *NLRC5* WT cases.

### 3.6 *NLRC5* expression is highly correlated with intratumoral T‐cell markers

**FIGURE 5:**
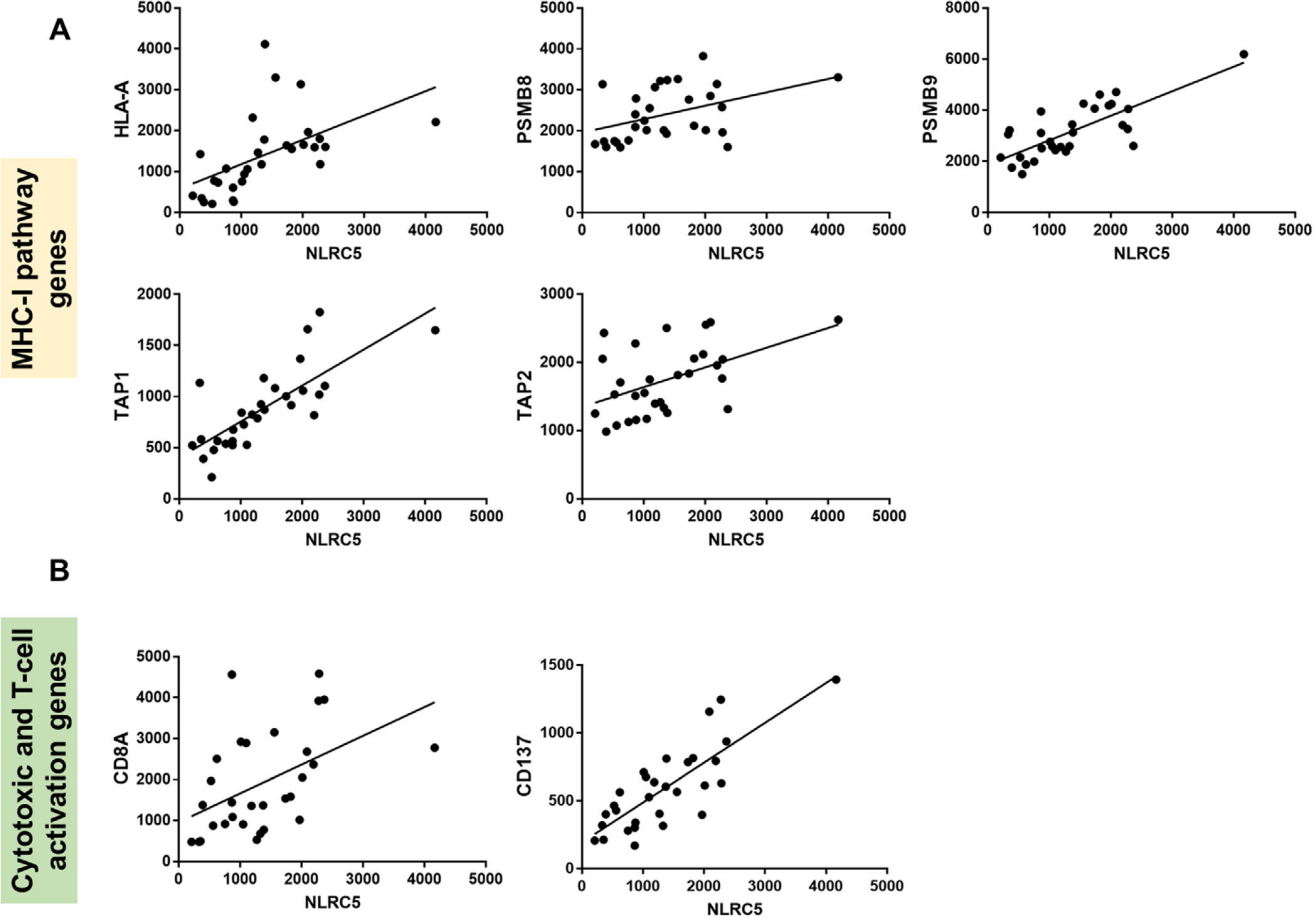
Gene expression correlations of *NLRC5* (A) with MHC‐I pathway and (B) with cytotoxic T‐cell (*CD8A*) and T‐cell activation (*CD137*) genes. All correlations *r *> 0.7, *P *< .0001

The relationship between *NLRC5*, MHC‐I genes, the cytotoxic T‐cell marker CD8, and the T‐cell activation marker CD137 was also interrogated. *NLRC5* gene expression highly correlated with *HLA‐A, PSMB8, PSMB9, TAP1*, and *TAP2* genes of the MHC‐I pathway (*r *> 0.7; *P *< .0001, Figure 5A), and with *CD8* and *CD137* consistent with NLRC5 having a pivotal role in the activation of CD8 T‐cells within these tumours (*r *> 0.7; *P *< .0001, Figure 5B).

### 3.7 *NLRC5* aberrations lead to reduced NLRC5 protein in FL tumours

**FIGURE 6:**
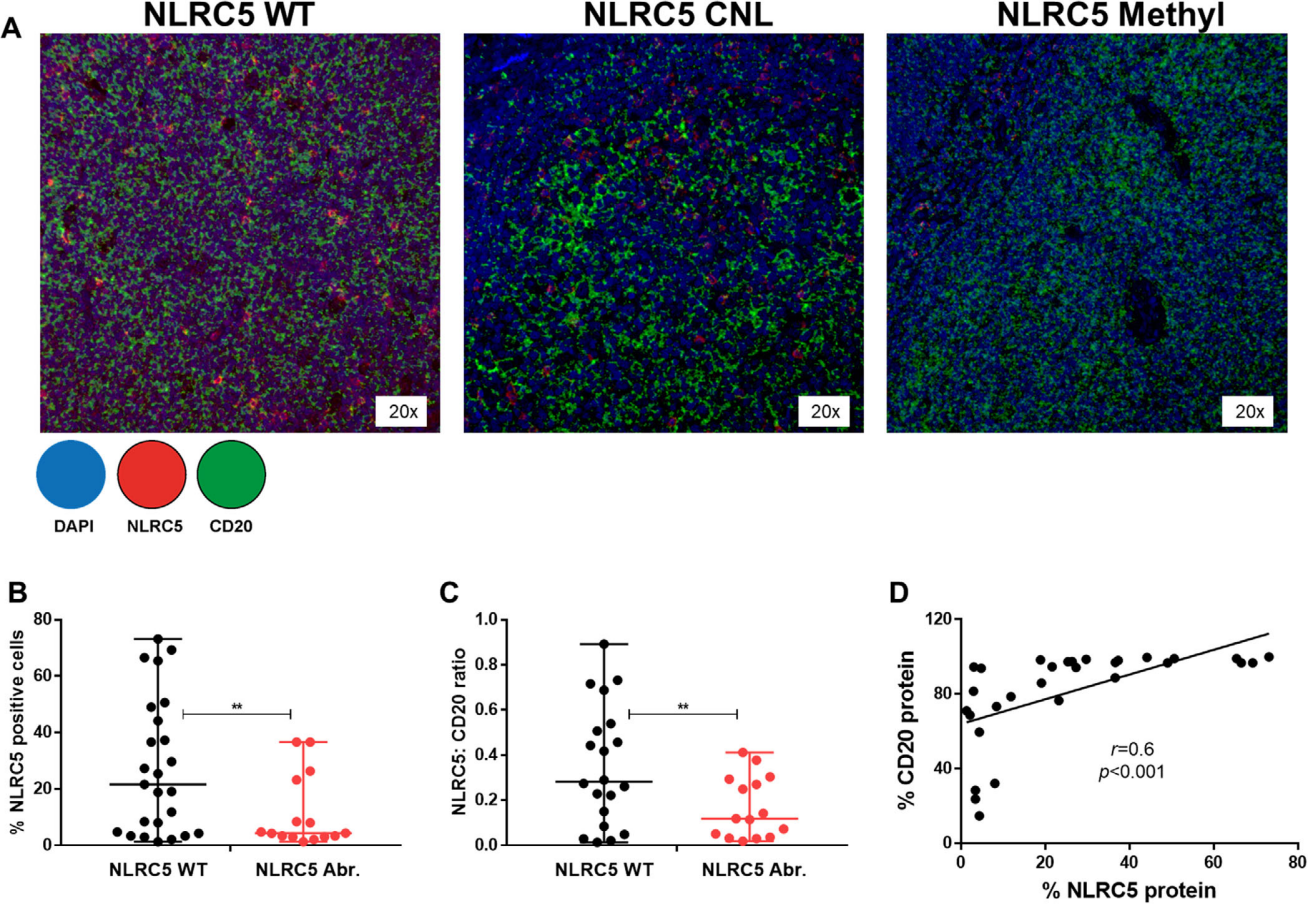
NLRC5 protein expression in FL tumours stratified by *NLRC5* status. A, Representative multispectral microscopic visualisation of NLRC5 (red) and CD20 (green) with 4′ 6‐diamidino‐2‐phenylindole (DAPI; dark blue) as the nuclear marker. B, Mean percentage of NLRC5‐positive cells. C, NLRC5:CD20 ratio and D, correlation of NLRC5 and CD20 protein expression in *NLRC5* WT (four tissue sections) and in *NLRC5* Abr. (three tissue sections) determined by acquisition of five random regions‐of‐interest for each sample at 20x resolution. WT, wild‐type; *NLRC5* Abr., *NLRC5* copy number lost/promotor methylated cases. **P < .01. Error bars, mean with SD.

Finally, we investigated if *NLRC5* aberrations affect NLRC5 protein expression in FL tumours. Using high‐resolution multiplexed multicolour immune fluorescent microscopy on tissue sections stratified by *NLRC5* aberrant status (Figure 6A), a lower percentage of NLRC5‐expressing cells were present in *NLRC5* aberrant compared to *NLRC5* WT cases (mean 11.3% vs 27.4%; *P *< .01, Figure 6B). Costaining with NLRC5 and CD20 antibodies confirmed that the decrease in NLRC5 seen in aberrant sections is attributed to B‐cells in these tumours (mean ratio 0.16% vs 0.35% in WT sections; *P *< .01, Figure 6C). In all tissue sections, NLRC5 protein expression highly correlated with CD20 protein expression (*r *> 0.6; *P *< .001, Figure 6D).

### 3.8 *NLRC5* gene aberrations do not appear to alter outcome after conventional frontline immunochemotherapy

Finally, we evaluated if harbouring *NLRC5* aberrations affects prognosis in patients treated by conventional frontline immunochemotherapy‐based treatment modalities. We found no survival advantage (OS and PFS) nor differences in treatment outcomes (FFS and TT2T) between patients with *NLRC5* aberrations compared to patients who are WT for *NLRC5* (Figure S3A). Similarly, no differences were observed between patients with all MHC‐I pathway aberrations combined compared to patients who have no aberrations (Figure S3B).

## 4 DISCUSSION

Immune evasion is a hallmark of cancer [29]. The mechanisms underpinning MHC‐I/CD8^+^ T‐cell axis disruption have been established in aggressive lymphoma [18, 19]. Although it was recently established that FL patients who go on to experience a POD24 event have reduced levels of intratumoural CD8^+^ T‐cell clonal expansion [23], no comprehensive analysis of the MHC‐I pathway in FL has been undertaken to date. In the current study, targeted sequencing in a cohort of 172 FL tumours identified abnormalities in NLRC5 as the most frequent gene abnormality observed in MHC‐I pathway genes. Hypermethylation of the *NLRC5* promoter was the most frequently observed *NLRC5* gene aberration and was mutually exclusive to CNL. Aberrations in *NLRC5* (CNL and hypermethylation combined) were associated with reduced NLRC5 RNA and protein expression and correlated with downregulation of MHC‐I antigen presentation and CD8^+^ T‐cell effector function.

Nucleotide‐binding domain and leucine‐rich repeats containing (NLR) proteins play an evolutionarily conserved role in host defense by functioning as sensors for pathogen‐derived microbe‐associated molecular patterns and danger‐associated molecular patterns [30, 31]. In addition to their role in innate immunity, nuclear‐translocated NLRs can act as transcriptional transactivators. The most studied member of this latter function is CIITA, which acts as a global regulator for constitutive and inducible expression of MHC‐II genes and their accessory genes. *CIITA* mutations, gene fusions, and translocations are well characterised in many NHLs [15, 32], including in FL [33]. Our study is the first to report copy number aberrations and promotor methylation abnormalities in the MHC‐I transactivator NLRC5, in any lymphoid malignancy.

To our knowledge, data on *NLRC5* CNL and promoter methylation in diffuse large B‐cell lymphoma (DLBCL) remain to be established. However, Morin et al have reported a single nonsense mutation (p.Q597*) in a DLBCL cohort of 276 tumours that was confirmed as somatic [34]. The functional consequence of this mutation remains unknown. Notably, in our FL cohort, *NLRC5* SNVs were present in a small number of cases but they were all germline SNVs that did not affect gene expression. Although further investigation across subtypes is required to definitively establish if somatic *NLRC5* mutations are limited to aggressive lymphomas, the lack of somatic mutations in FL that we observed suggests this is not a major mechanism of MHC‐I‐mediated immune evasion in indolent lymphoma.

**FIGURE 7:**
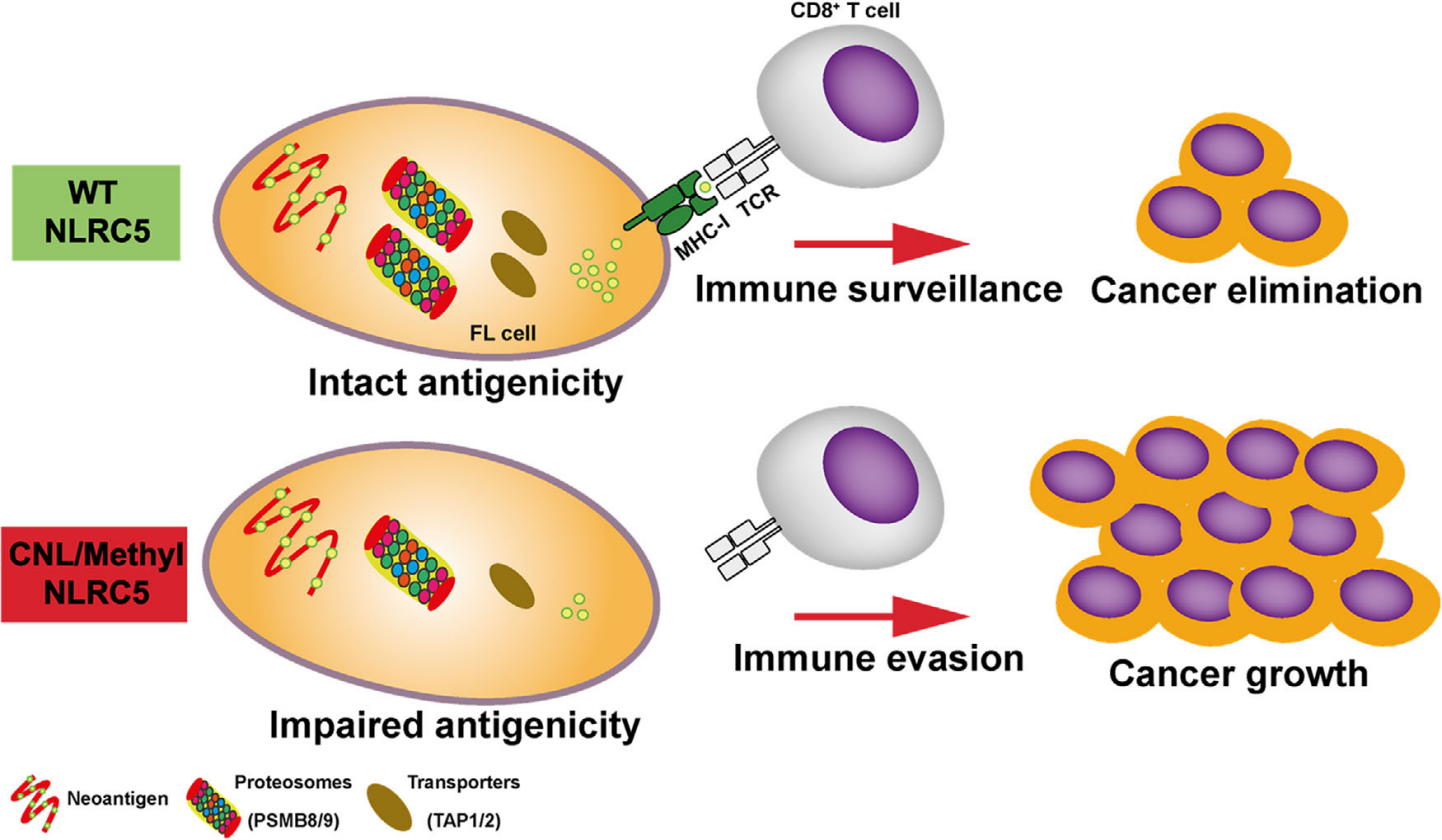
Schematic model of how NLRC5 loss leads to reduced antigenicity and host immune evasion. Genetic and epigenetic aberrations in *NLRC5* diminish MHC‐I surface expression. The subsequent reduction in antigen‐presentation impairs CD8^+^ T‐cell activation. These changes result in an impaired ability to elicit antitumour CD8^+^ T‐cell responses and reduced CD8^+^ T‐cell infiltration in cancer tissues.

To avoid elimination, tumours use immune‐evasion strategies to establish an immune‐suppressive microenvironment. Broadly, this is achieved by tumour cells either losing immunogenicity whereby immunoinhibitory molecules are upregulated by the tumour and/or the tumour microenvironment, or by establishing impaired antigenicity in which malignant cells lose their ability to be recognised by the host immune system [35]. Loss of antigen presentation prevents the priming of naïve tumour‐specific T‐cells and also the recognition of tumour cells by antigen‐experienced T‐cells, both of which lead to tumours becoming invisible to antitumour cytotoxic T‐cells (Figure 7) [36]. Indeed, *Nlrc5*‐deficient mice exhibit profound MHC‐I disruption and concomitant reduced CD8^+^ T‐cell levels and activity [37, 38]. Similarly, Rodriguez et al showed that B16 melanoma cell lines stably expressing NLRC5 re‐established MHC‐I antigen presentation and induced the activation and proliferation of Pmel‐1 TCR transgenic CD8^+^ T‐cells in response to endogenous tumour antigen. Upon subcutaneous transplantation of NLRC5‐expressing cells, recipient mice showed markedly reduced tumour growth demonstrating that NLRC5 could be exploited to elicit antitumour immunity [39]. Furthermore, exploitation of the NLRC5 pathway may have therapeutic potential. For example, patient‐derived tumour cells can be genetically engineered to overexpress NLRC5 *in vitro*. This would facilitate the identification of private tumour epitopes, and endogenous antitumour T‐cell clones can be expanded accordingly [40].

Current predictive biomarkers of cancer immunotherapy focus on immunogenicity, such as expression of PD‐L1 molecules [41]. Lymphomas show marked differential sensitivity to CPI [42] and this is likely to be at least in part a result of impaired antigenicity of the malignant B‐cells. It is unlikely that treatment with CPIs alone will have any effect on these tumours. Intact *NLRC5* and its ancillary genes could be used as potential genetic biomarkers to pre‐test patients before immunotherapy to clearly identify patients with reduced antigenicity and hence would be less likely to benefit from CPI. These patients might be better considered for alternate treatment options.

In summary, our data establish NLRC5 as a novel MHC‐I‐related immune evasion mechanism utilised by FL tumours to establish impaired antigenicity. Further studies are required to investigate strategies to circumvent MHC‐I‐mediated immune evasion, and also to determine whether patients with aberrant *NLRC5* have a diminished response to immunotherapy.

## AUTHORSHIP CONTRIBUTIONS

J.G. conceived the project, designed and performed the research, analysed and interpreted data, and co‐wrote the manuscript. J.N.L., K.B., L.M.L. and M.B.S. performed experiments and interpreted data. V.M performed bioinformatic analyses of sequencing data. S.B. and J.W.D.T. curated the study cohort and acquired clinical data. M.K.G. oversaw the project, designed the research, interpreted data, and co‐wrote the manuscript. All authors approved the final version.

## CONFLICT OF INTEREST

The authors declare no competing financial interests.

## DATA SHARING

The data that support the findings of this study are available from the corresponding author upon reasonable request.

## Supporting information

Figure S1. Validation of *NLRC5* SNVs by Sanger sequencing.

Figure S2.

Figure S2. Targeted NLRC5 sequencing in FL tumors and in matched peripheral blood samples. (A) Representation of NLRC5 coding sequence SNVs in 172 patient tumors found by targeted sequencing. (B) Sanger sequencing of four representative FL tumors and their matched peripheral blood confirming the SNVs are germline. (C) NLRC5 gene expression in FL tumors stratified by NLRC5 genetic status. NLRC5 gene expression is not reduced by germline variation (SNV). NS, not significant. Error bars, mean with SD.

Figure S3. Kaplan‐Meier survival analysis of FL patients stratified by (A) *NLRC5* and (B) MHC‐I pathway gene status. WT, wild‐type; *NLRC5* Abr., *NLRC5* copy number lost/promotor methylated cases; MHC‐I Abr., cases with *NLRC5* copy number loss/promotor methylation and cases with MHC‐I pathway SNVs and copy number alterations; OS, overall survival; PFS, progression‐free survival; FFS, failure‐free survival; TT2T, time to second treatment; NS, not significant.
