# Supplementary figures and images for: Genetic aberrations of NLRC5 are associated with downregulated MHC‐I antigen presentation and impaired T‐cell immunity in follicular lymphoma
Source: EJHaem. 2020 Oct 14;1(2):517–26. doi: 10.1002/jha2.116 (PMC9176136; doi:10.1002/jha2.116)

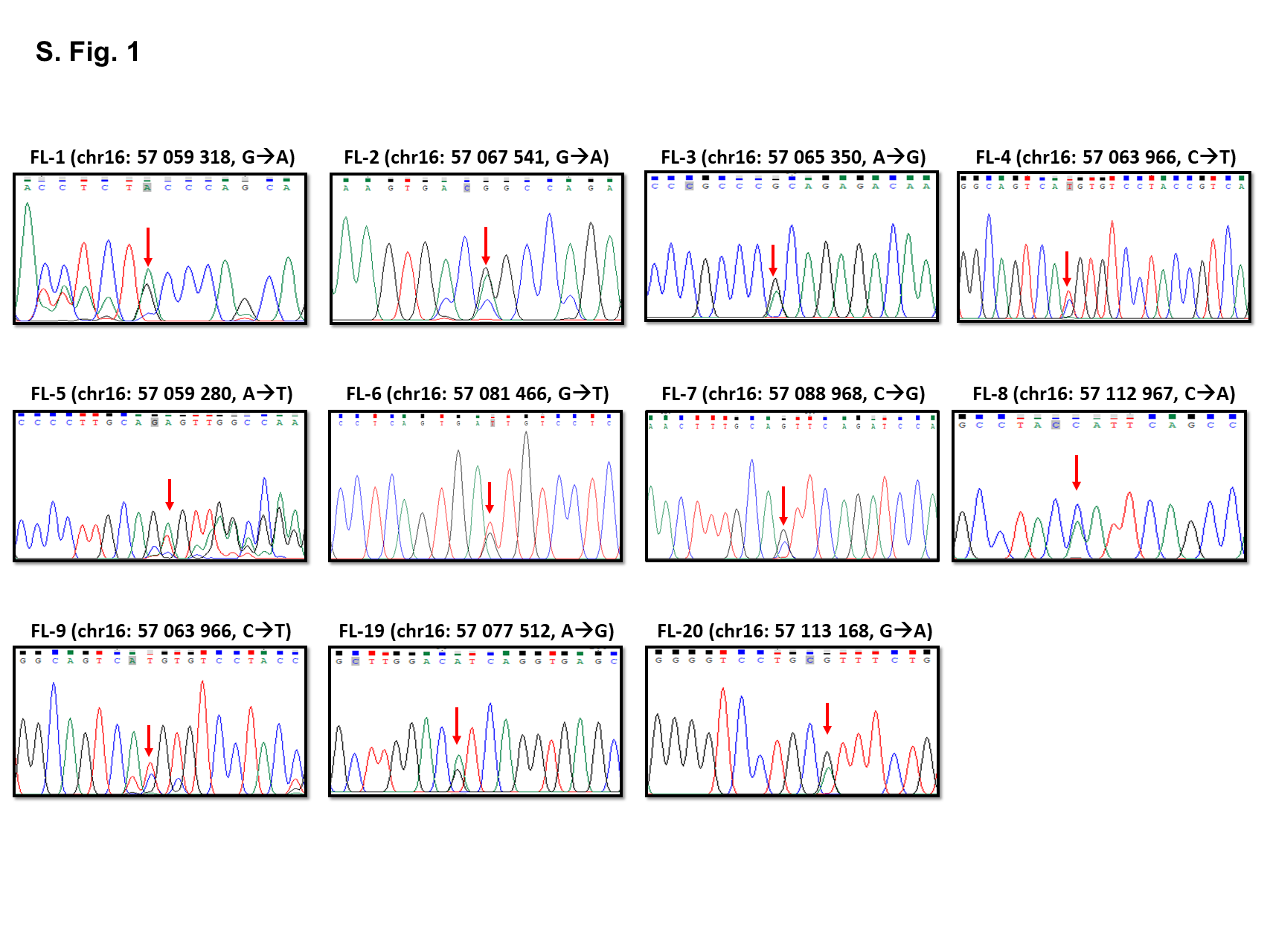

Supplement: Supplementary file 1 — Figure S1. Validation of NLRC5 SNVs by Sanger sequencing. [file JHA2-1-517-s003.tif]

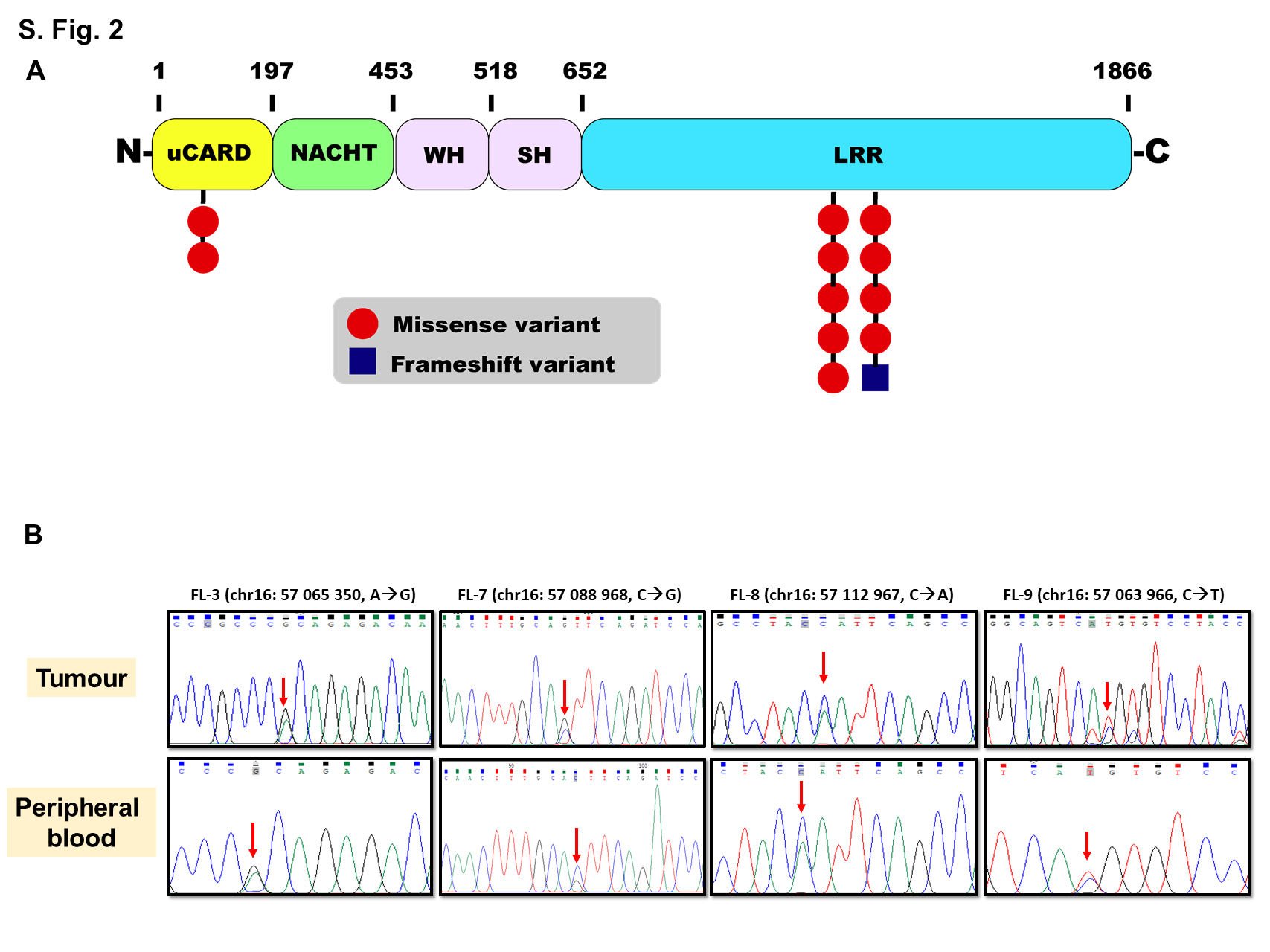

Supplement: Supplementary file 2 — Figure S2. [file JHA2-1-517-s004.tif]

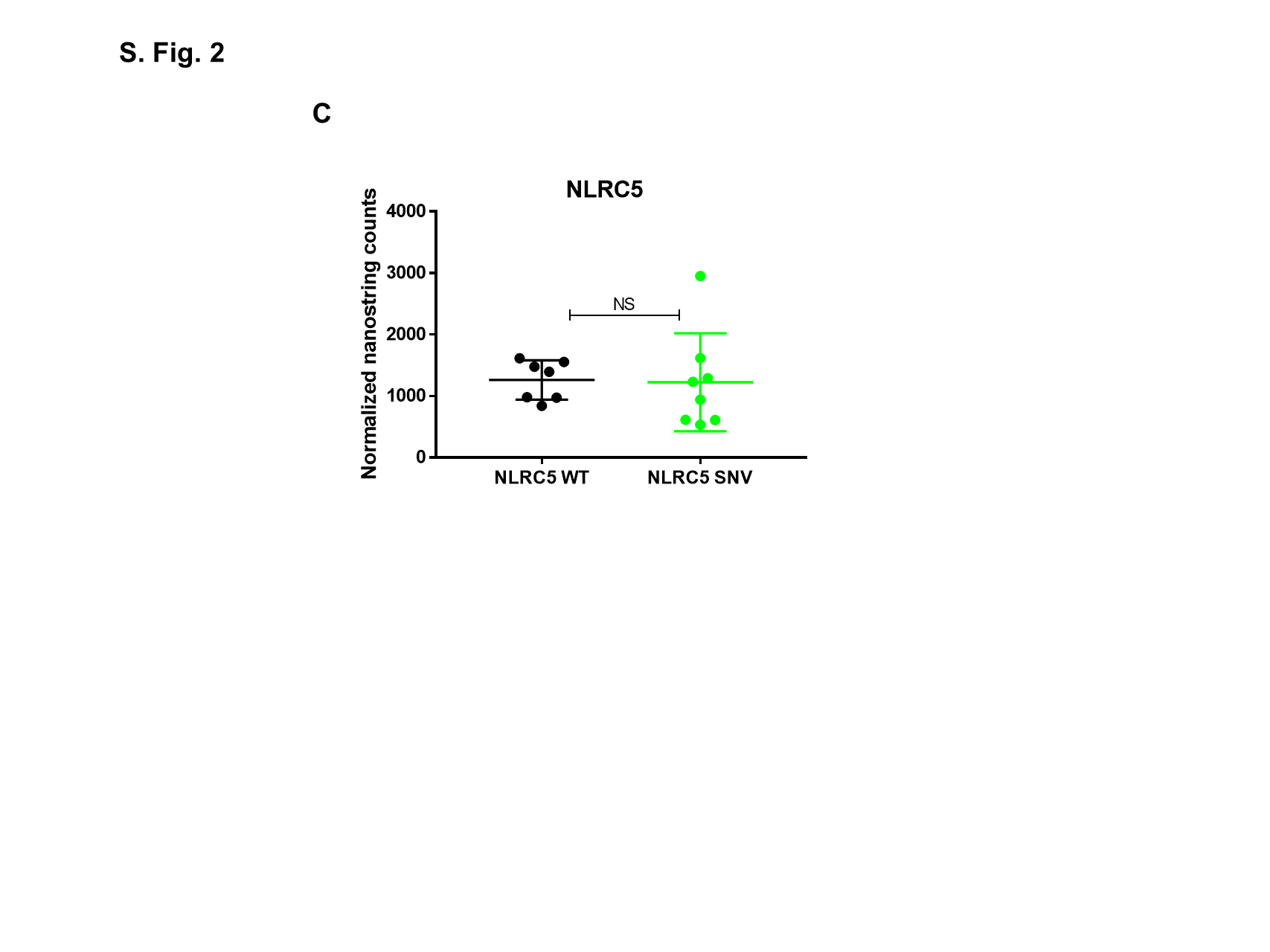

Supplement: Supplementary file 3 — Figure S2. Targeted NLRC5 sequencing in FL tumors and in matched peripheral blood samples. (A) Representation of NLRC5 coding sequence SNVs in 172 patient tumors found by targeted sequencing. (B) Sanger sequencing of four representative FL tumors and their matched peripheral blood confirming the SNVs are germline. (C) NLRC5 gene expression in FL tumors stratified by NLRC5 genetic status. NLRC5 gene expression is not reduced by germline variation (SNV). NS, not significant. Error bars, mean with SD. [file JHA2-1-517-s002.tif]

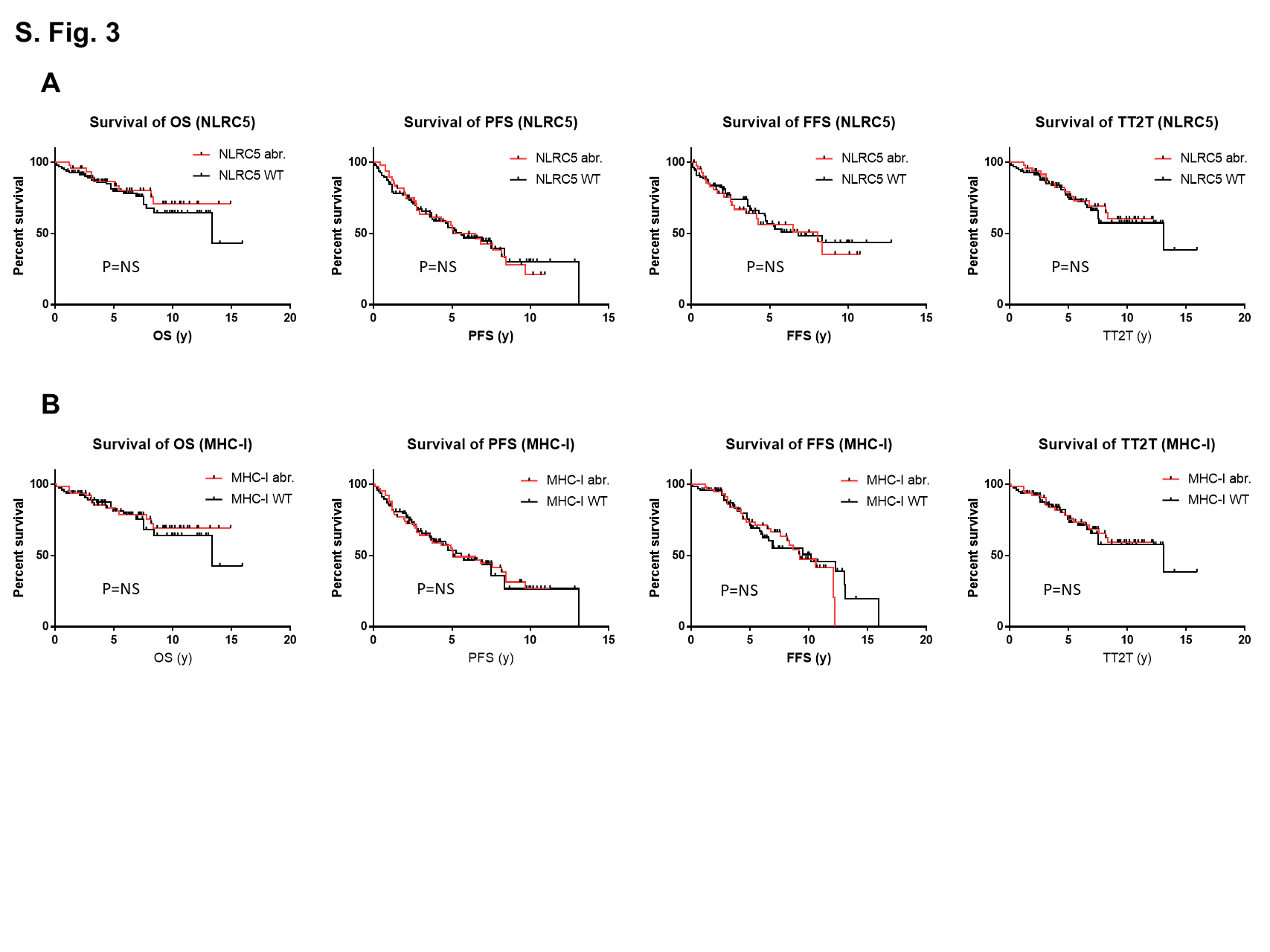

Supplement: Supplementary file 4 — Figure S3. Kaplan‐Meier survival analysis of FL patients stratified by (A) NLRC5 and (B) MHC‐I pathway gene status. WT, wild‐type; NLRC5 Abr., NLRC5 copy number lost/promotor methylated cases; MHC‐I Abr., cases with NLRC5 copy number loss/promotor methylation and cases with MHC‐I pathway SNVs and copy number alterations; OS, overall survival; PFS, progression‐free survival; FFS, failure‐free survival; TT2T, time to second treatment; NS, not significant. [file JHA2-1-517-s001.tif]
